# A new species of blind snake, *Indotyphlops* (Serpentes, Typhlopidae), from the Qinghai-Xizang Plateau, China

**DOI:** 10.3897/zookeys.1292.197322

**Published:** 2026-09-17

**Authors:** Bing Lyu, Songwen Tan, Zhichao Zhao, Van Wallach, Qin Liu, Peng Guo

**Affiliations:** 1 Faculty of Agriculture, Forestry and Food Engineering, Yibin University, Yibin 644005, China Faculty of Agriculture, Forestry and Food Engineering, Yibin University Yibin China; 2 Yibin Key Laboratory of Zoological Diversity and Ecological Conservation & Yibin Inspection and Quarantine Engineering Technology Research Center of Animal and Plant, Yibin University, Yibin 644005, China Yibin Key Laboratory of Zoological Diversity and Ecological Conservation & Yibin Inspection and Quarantine Engineering Technology Research Center of Animal and Plant, Yibin University Yibin China; 3 4 Potter Park, Cambridge, MA 02138, USA Unaffiliated Cambridge United States of America; 4 College of Life Science, Sichuan Normal University, Chengdu 610101, China College of Life Science, Sichuan Normal University Chengdu China

**Keywords:** Blind snake, morphology, new species, Qinghai-Xizang Plateau, taxonomy, Typhlopidae

## Abstract

Typhlopidae is the most species-rich and broadly distributed family of blind snakes. Due to their secretive habits, fossorial lifestyle, and conserved morphology, the taxonomy and systematics of this group remain poorly understood. Here, a new species of blind snake is described from Xizang, China, based on morphological comparisons and molecular phylogenetic analyses. The new species is genetically divergent from its closest relatives with an uncorrected CO1p-distance exceeding 18.3%. Morphologically, it can be diagnosed by a combination of the following characters: 1) large size with 265 mm total length, 2) supralabial imbrication pattern T-V, 3) midbody scales in 18 rows, 4) total middorsal scales 432, 5) subcaudals 17, 6) infralabials 2, 7) absence of a terminal tail spine, and 8) light-colored head cap. Currently, the new species is known only from Xizang, China, but it likely occurs in adjacent regions in Nepal.

## Introduction

Blind snakes (Scolecophidia) are an ancient group of snakes classified as the superfamily Typhlopoidea and characterized by their fossorial lifestyle and highly specialized morphological features. Presently, five families are recognized in this group (Uetz et al. 2026): Anomalepididae, Gerrhopilidae, Typhlopidae, Leptotyphlopidae, and Xenotyphlopidae. Of these, Typhlopidae is the most species-rich with approximately 280 species (Uetz et al. 2026). Despite their wide distribution and species diversity, the blind snakes in this family are often overlooked in scientific literature, and their systematics and phylogenetics remain poorly understood. In recent decades, new species have been successively described in many places, such as China (Wallach and Pauwels 2004), Thailand (Wallach 2021), Sri Lanka (Wickramasinghe et al. 2023), and Timor-Leste (O’Shea et al. 2023), suggesting that its species diversity may be underestimated and emphasizing the urgency of biodiversity studies.

The Qinghai-Xizang Plateau (QXP), an extensive inland plateau in China, is the largest plateau in the country and the highest in the world. Widely known as the “Roof of the World” and the “third pole of the earth,” it stands as a critical hotspot for global biodiversity and evolutionary research. Characterized by unique ecosystems encompassing diverse habitats, varied climates, and complex geological and geomorphological types, the QXP harbors an exceptionally rich species diversity (Wang et al. 2024; Guo et al. 2024, 2025; Tan et al. 2026; Liu et al. 2026). This region also hosts a remarkable assemblage of endemic reptiles and amphibians, including snakes (e.g., *Thermophis* sp., *Protobothrops xiangchengensis*, and *Gloydius strauchi*) (Guo and Che 2024), lizards (e.g., *Scincella jinchuanensis* and *Diploderma batangense*) (Liu et al. 2026; Che and Wang 2026), frogs (e.g., *Amolops xinduqiao*) (Che and Lu 2026). Additionally, two blind snake species, *Argyrophis diardii* and *Indotyphlops braminus*, have been recorded to occur in the Hengduan Mountains, the eastern sector of the QXP (Guo and Che 2024).

In 2025, a single blind snake specimen was collected from the QXP of China, in the vicinity of the China-Nepal border. Combined analyses from molecular phylogenetic analyses and morphological comparisons consistently suggest that this specimen represents a new species, which is therefore described below.

## Materials and methods

### Molecular analyses

**Figure 1.**
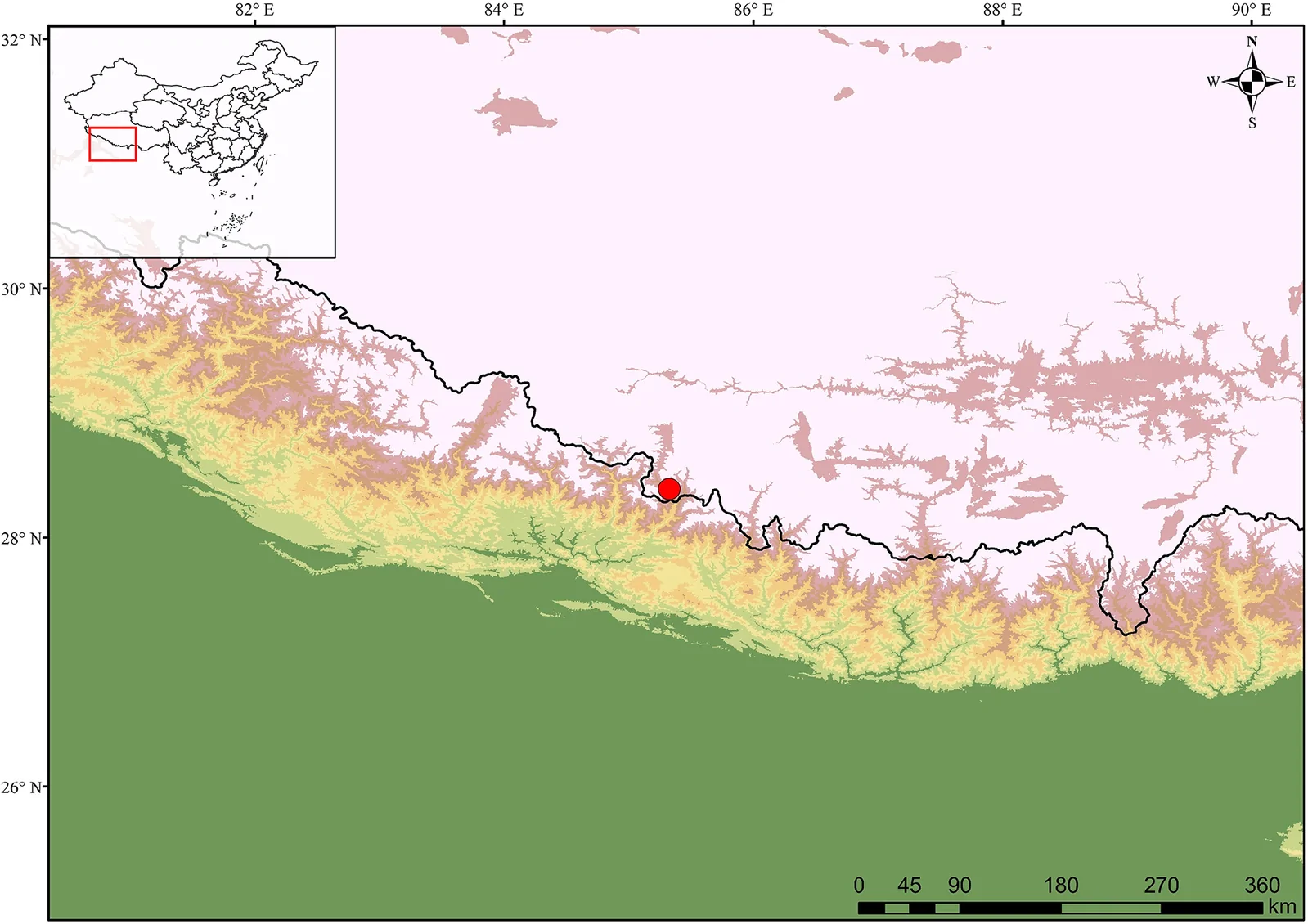
Map showing the locality of the new species in Gyirong, Xizang, China.

One blind snake specimen was collected during fieldwork in Gyirong, Xizang, China (Fig. 1). After euthanasia, a fresh liver tissue sample was taken, preserved in 90% ethanol, and stored at −80 °C. The specimen was fixed in 10% formalin and subsequently transferred to 75% ethanol for permanent storage at room temperature. Both the tissue sample and specimen were deposited at Yibin University (YBU), China.

Genomic DNA was extracted from the liver tissue using the TIANamp Genomic DNA Kit (TIANGEN Bio-tech Co., Ltd., Beijing, China) following the manufacturer’s protocols. Partial fragments of the mitochondrial cytochrome *b* (Cytb) and cytochrome *c* oxidase subunit 1 (CO1) genes were amplified and sequenced using the primers 14910/16064 (Burbrink et al. 2000) and Chmf4/Chmr4 (Che et al. 2012), with cycling parameters identical to those described in the above studies. Polymerase chain reaction (PCR) products were purified and bidirectionally sequenced by Sangon Biotech Co., Ltd (Chengdu, China).

The generated sequences were manually edited using SeqMan in Lasergene ver. 7.1 (DNASTAR, USA), aligned using the ClustalW algorithm with default parameters in MEGA ver. 7.0 (Kumar et al. 2016), and visually inspected for minor misalignments, which were manually adjusted. The DNA sequence was quality-checked in MEGA ver. 7.0 for any unexpected stop codons. The sequences were then integrated into the dataset from Pyron and Wallach (2014), encompassing 97 species and 10 genes (four mitochondrial and six nuclear) (Suppl. material 1).

Three representatives of the Gerrhopilidae and Xenotyphlopidae families were included in the dataset, and one individual was used as the outgroup. Bayesian inference (BI) was employed to reconstruct the phylogenetic relationships within this group using MrBayes ver. 3.2.2 (Ronquist et al. 2012). The best-fit nucleotide substitution model for each codon position (coding genes) and genes (non-coding genes) was the same as that of Pyron and Wallach (2014). Analyses were performed with three independent runs, each initiated with a random tree and four Markov chains (three heated and one cold). Sampling was performed every 2000 generations over 10 × 10^6^ generations, with the first 25% of samples discarded as burn-in after convergence assessment in Tracer ver. 1.7 (Rambaut et al. 2018). The resulting trees were combined to determine the posterior probabilities (PP) for each node using a 50% majority-rule consensus tree.

Genetic distances between species were calculated in MEGA ver.7.0 using the uncorrected pairwise genetic distance (p-distance) (Kumar et al. 2016).

### Morphological examination

The specimen was morphologically examined following the methodology described in Zhao et al. (1998) and Zhao (2006). Body and tail lengths were measured to the nearest 1.0 mm using an inelastic ruler, and symmetric meristic characters were examined bilaterally (left/right) under a stereomicroscope. Morphological data for related species were sourced from previous publications (Hedges et al. 2014; Pyron and Wallach 2014; O’Shea et al. 2023; Wickramasinghe et al. 2023).

## Results

### Molecular phylogeny

**Figure 2.**
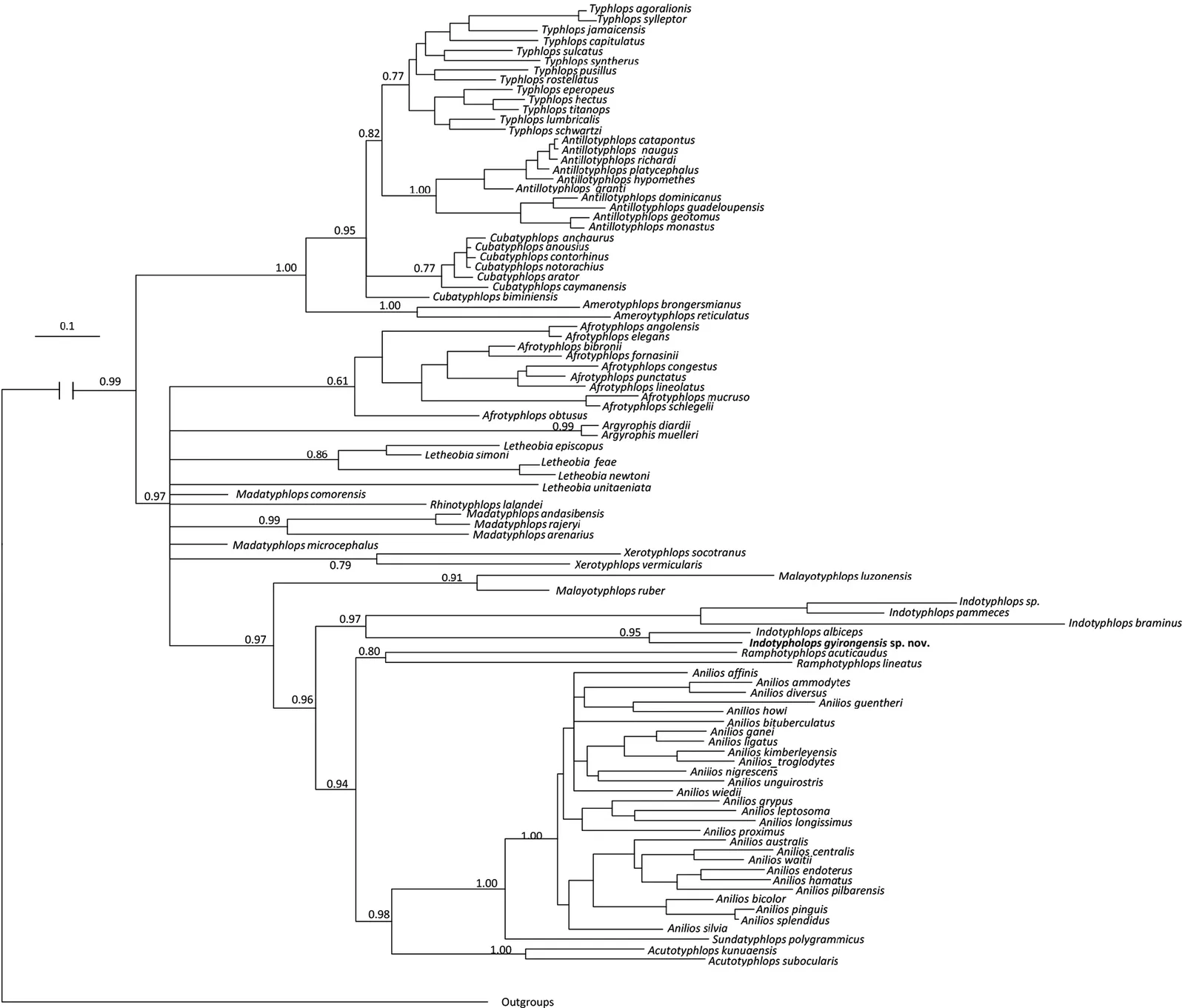
Bayesian majority-rule consensus tree of *Indotyphlops* inferred from the combined data using models detailed in the text. Posterior probabilities from Bayesian inference (> 0.50) are given adjacent to respective major nodes. Branch support indices are not given for most nodes to preserve clarity.

The final alignment was 3128 base pairs (bps) in length. BI analyses yielded a phylogenetic tree nearly identical to that of Pyron and Wallach (2014). The newly collected sample from Gyirong County, Xizang, China, was nested within the *Indotyphlops* clade and formed a well-supported monophyletic lineage with its congeners (PP = 0.97). Within this genus, this sample was recovered as sister to *I. albiceps* (Boulenger, 1898), with strong support (PP = 0.95) (Fig. 2).

For the mitochondrial Cytb gene, the uncorrected p-distance between the novel specimen and its phylogenetically closest congener with publicly available sequences exceeded 24% (for the undescribed *Indotyphlops* sp.), while for the CO1 gene, this value reached 18.3% (for *I. braminus* (Daudin, 1803)) (Suppl. materials 2, 3). The two newly generated sequences were submitted to GenBank (PZ862014 and PZ339874) (Suppl. material 1).

### Morphological examination

Detailed morphological examination showed that the new specimen fell within the genus *Indotyphlops* based on total body length, body scales, and middorsal numbers (see details below). It also differs from other species in this genus by various characters, including midbody scales, head cap, and supralabial imbrication pattern (SIP) (see below).

### Taxonomy

#### Indotyphlops gyirongensis sp. nov.

Taxon classificationAnimaliaSquamataTyphlopidae

06E763D7-4D16-5004-AFD5-07F6090D8EB7

https://zoobank.org/10D817CB-1397-4D2A-B929-2DC30F1B3E88

Figs 3, 4, 5

##### Holotype.

• YBU 251031, adult female collected from Gyirong Town, Gyirong County, Xizang, China (28.31°N, 85.33°E), at an elevation of 2262 m in August 2025.

**Figure 3.**
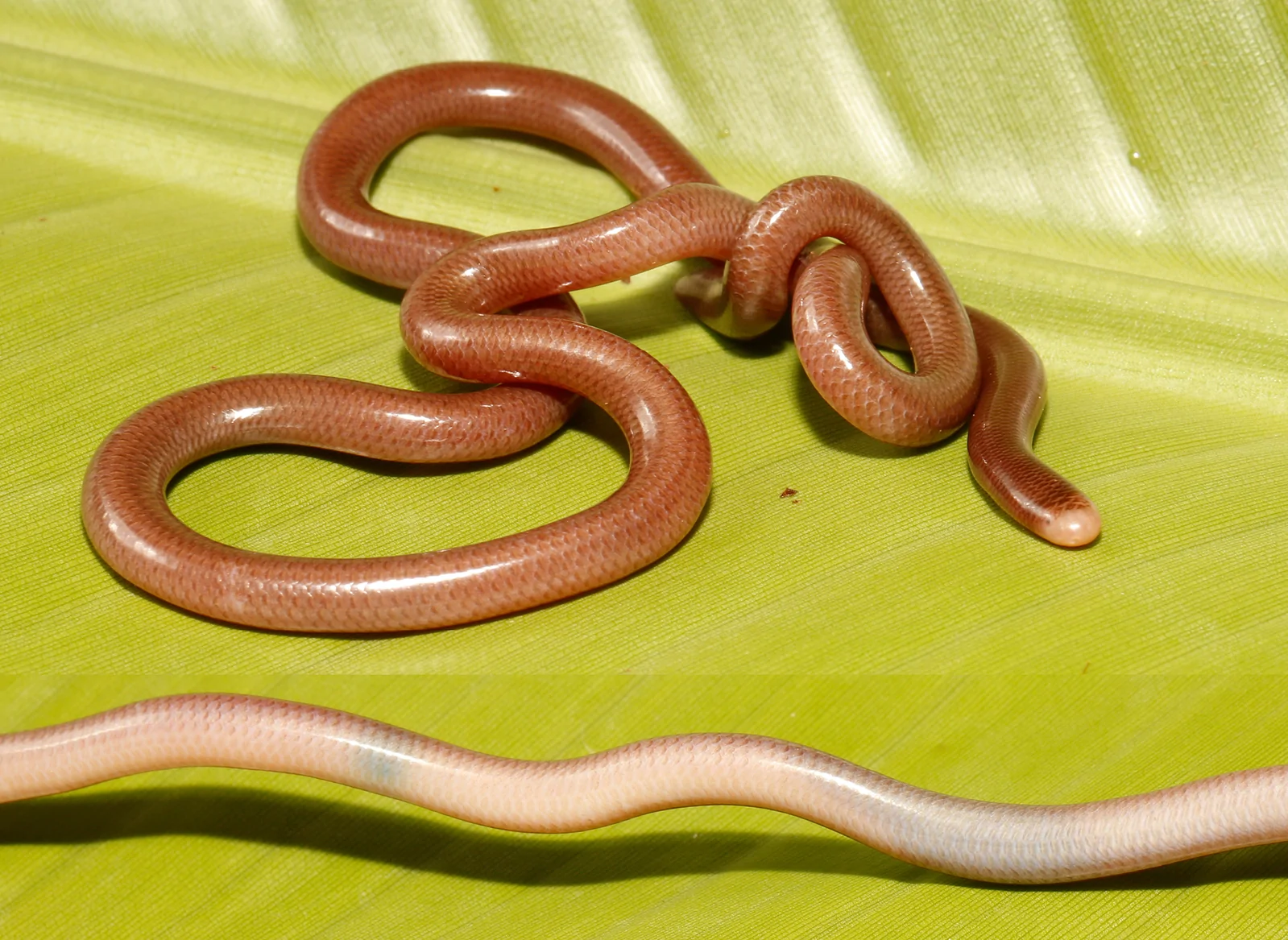
General (top) and ventral (below) views of the holotype of *Indotyphlops gyirongensis* sp. nov. in life (by Peng Guo).

**Figure 4.**
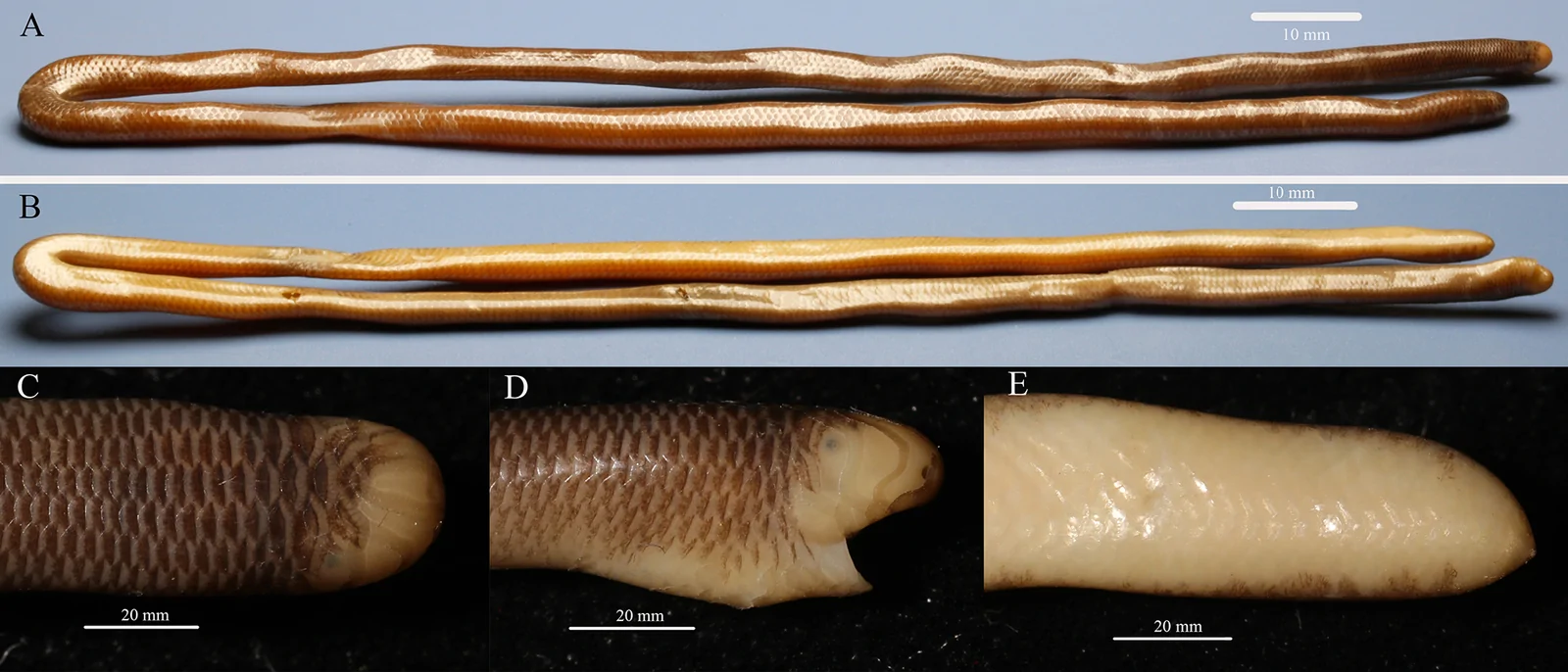
Holotype of *Indotyphlops gyirongensis* sp. nov. in preservative. Dorsal (**A**) and ventral (**B**) views of the body, dorsal (**C**) and lateral (**D**) views of head, and ventral view of tail (**E**) (by Peng Guo).

**Figure 5.**
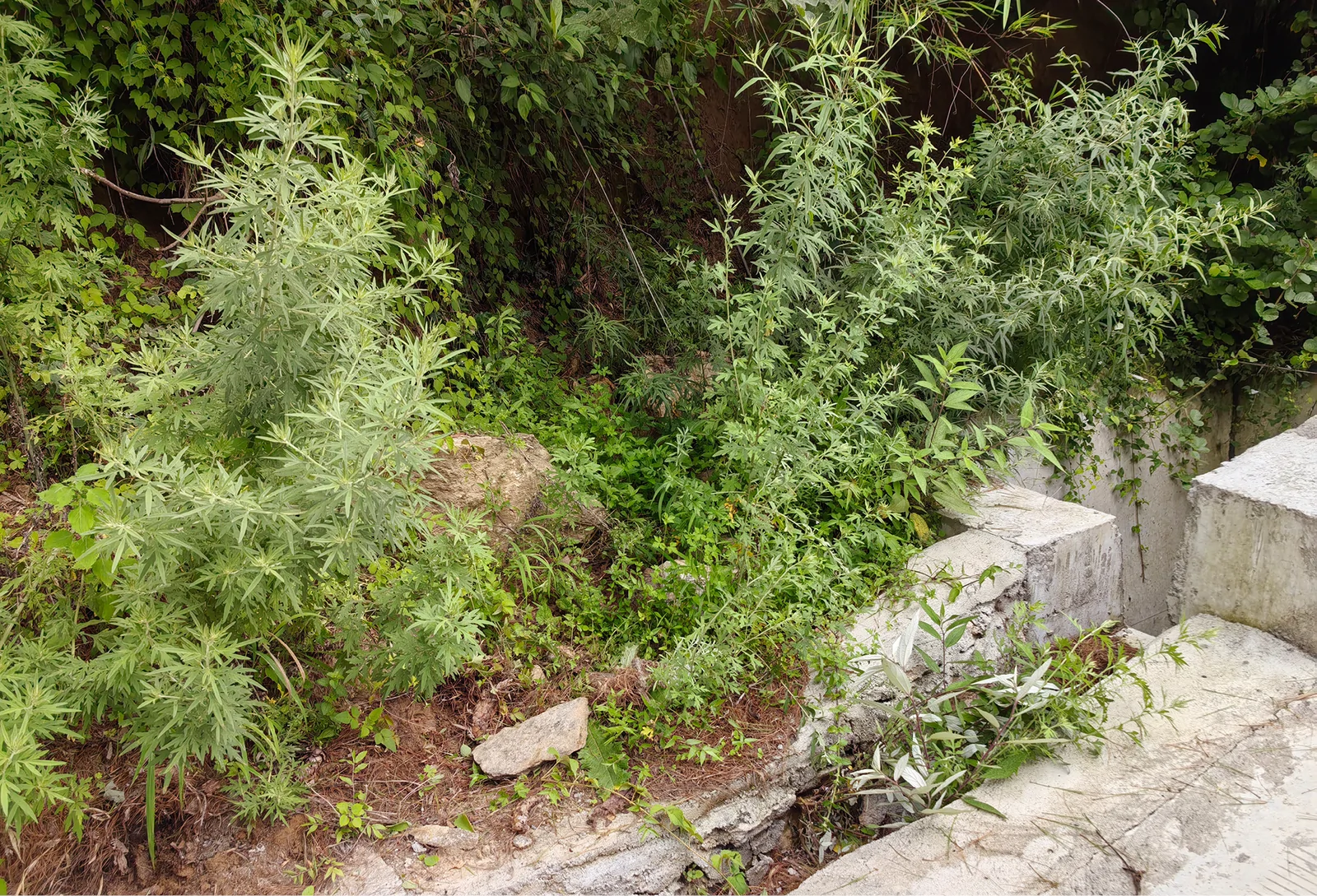
Microhabitat of the type specimen of *Indotyphlops gyirongensis* sp. nov. in Gyirong, Xizang, China (by Peng Guo).

##### Diagnosis.

A blind snake distinguished from congeners by a combination of characters: 1) large size with 265 mm in total length, 2) SIP T-V, 3) midbody scales in 18 rows, 4) total middorsal scales 432, 5) subcaudals 17, 6) infralabials 2, 7) absence of a terminal tail spine, and 8) light-colored head cap.

##### Description of the holotype.

A slender female blind snake with total length (TL) of 265 mm and tail length (TaiL) of 7.5 mm (TaiL/TL = 2.8%), midbody diameter (BD) of 3.5 mm (TL/BD = 76), midtail diameter (TD) 3.4 (TaiL/TD = 2.2); dorsal scale row formula 18-18-18, uniform in size, smooth and glossy; total middorsals 432, subcaudals 17; tail lacking spine but terminating in a nubbin.

Head rounded in dorsal profile, indistinct from body; rostral ogival in shape, approximately 1/3 the width of midocular level of head (RW/HW = 0.35); frontal and postfrontal transversely enlarged; supraoculars oblique, slightly narrower than frontal; parietals and occipitals slightly broader than frontal and transversely oriented. Head bluntly rounded in lateral profile; nasal semidivided, nostril semilunar in shape and vertically oriented, inferior nasal suture contacting 2^nd^ supralabial; superior nasal suture extending 2/3 nasal-rostral distance; preocular single, 1.5 times the width of the nasal; ocular slightly narrower than preocular, dark eyespot present, lacking pupil and iris; postoculars 2; supralabials 4, SIP T-V, with 2^nd^ supralabial overlapping the preocular and 3^rd^ supralabial overlapping the ocular (see Pyron and Wallach 2014 for definition); infralabials 2.

In coloration, it is distinguished by a light cream-colored head cap, lacking pigmentation posteriorly as far as the ocular and 4^th^ supralabial; dorsal coloration light brown, gradually fading towards the tail, each scale covered with dark pigmentation on the posterior half and lacking pigmentation on anterior half; venter and subcaudals cream, lacking pigmentation and partially transparent to internal viscera; lateral body region lacking discrete demarcation between dorsum and venter.

##### Comparisons.

Currently there are at least 20 recognized species in the genus *Indotyphlops*, including *I. combank* Wickramasinghe et al., 2023, *I. albiceps*, *I. braminus*, *I. exiguus* (Jan, 1864), *I. jerdoni* (Boulenger, 1890), *I. laca* (O’Shea et al., 2023), *I. lankaensis* (Taylor, 1947), *I. lazelli* (Wallach & Pauwels, 2004), *I. leucomelas* (Boulenger, 1890), *I. longissimus* (Dumeril & Bibron, 1844), *I. loveridgei* (Constable, 1949), *I. malcolmi* (Taylor, 1947), *I. meszoelyi* (Wallach, 1999), *I. pammeces* (Gunther, 1864), *I. tenebrarum* (Taylor, 1947), *I. veddae* (Taylor, 1947), *I. porrectus* (Stoliczka, 1871), *I. schmutzi* (Auffenberg, 1980), *I. tenuicollis* (Peters, 1864), and *I. violaceus* (Taylor, 1947) (Hedges et al. 2014; Pyron and Wallach 2014; Uetz et al. 2026). Uetz et al. (2026) recognize *I. ahsanai* (Khan, 1999), *I. fletcheri* (Wall, 1919), and *I. madgemintonae* (Khan, 1999). Hedges et al. (2014) also recognize *I. filiformis* (Dumeril & Bibron, 1844), *I. hypsibothrius* (Werner, 1917), *I. khoratensis* (Taylor, 1962), *I. madgemintonae* (Khan, 1999), and *I.* “*ozakiae*” (Wallach in Niyomwan, 1999). The new species is most similar to taxa with a T-V SIP pattern and 18 scale rows (*I. exiguus*, *I. laca*, *I. lazelli*, *I. porrectus*, and *I. schmutzi*).

The new species can be differentiated from its congeners by the following characters:

1) It differs from *I. albiceps* in its SIP (T-V vs. T-III) and fewer scale rows (18 vs. 20).

2) It differs from *I. braminus* in its SIP (T-V vs. T-III), fewer scale rows (18 vs. 20), its superior nasal suture (invisible dorsally vs. visible dorsally), its inferior nasal suture contact (preocular vs. supralabial 2), and a light-colored head cap (present vs. absent).

3) It differs from *I. combank* in having fewer scale rows (18 vs. 20), a larger body size (total length 265 mm vs. 131 mm), and a stouter body (TL/BD = 76 vs. < 57), its superior nasal suture (invisible dorsally vs. visible dorsally), having more postoculars (2 vs. 1), and a light-colored head cap (present vs. absent).

4) It differs from *I. exiguus* in having more middorsals (432 vs. 348) and a light-colored head cap (present vs. absent).

5) It differs from *I. jerdoni* in its SIP (T-V vs. T-III), having fewer midbody scale rows (18 vs. 20-22), by its superior nasal suture (invisible dorsally vs. visible dorsally), having more postoculars (2 vs. 1), in its eye (lacking pupil vs. distinct pupil present), and its light-colored head cap (present vs. absent).

6) It differs from *I. laca* in a larger body size (total length 265 mm vs. 121 mm), relative tail length (TaiL/TL = 2.8 vs. 1.6), and in its light-colored head cap (present vs. absent).

7) It differs from *I. lankaensis* in its SIP (T-V vs. T-III), having fewer midbody scale rows (18 vs. 20), having more total middorsals (432 vs. < 261), having a more gracile body (TL/BD = 76 vs. < 35), its lateral snout profile (rounded vs. obtuse), its superior nasal suture (invisible dorsally vs. visible dorsally), in the inferior nasal suture contact (preocular vs. supralabial 2), having more postoculars (2 vs. 1), and a light-colored head cap (present vs. absent).

8) It differs from *I. lazelli* in its larger body size (total length 265 mm vs. 158 mm), a longer tail (TaiL/TL = 2.8 vs. 1.8), and its light-colored head cap (present vs. absent).

9) It differs from *I. leucomelas* in its SIP (T-V vs. T-III), fewer midbody scale rows (18 vs. 22), more total middorsals (432 vs. < 325), a larger body size (TL 265 mm vs. 130 mm), a more gracile body (TL/BD = 76 vs. < 32), by its superior nasal suture (invisible dorsally vs. visible dorsally), its eye (lacking pupil vs. distinct pupil present), and its light-colored head cap (present vs. absent).

10) It differs from *I. longissimus* in its SIP (T-V vs. T-III), fewer midbody scale rows (18 vs. 22), its tail proportion (TaiL/TD = 2.2 vs. 1.0), its rostral width (RW/HW = 0.35 vs. > 0.48), its relative tail length (TaiL/TL = 2.8 vs. 1.5), and its light-colored head cap (present vs. absent).

11) It differs from *I. loveridgei* in its SIP (T-V vs. T-III), its tail proportion (TaiL/TD ratio 2.2 vs. 1.4), more postoculars (2 vs. 1), its eye (eyespot present vs. absent), and its light-colored head cap (present vs. absent).

12) It differs from *I. malcolmi* in its SIP (T-V vs. T-III), fewer midbody scale rows (18 vs. 20), more total middorsals (432 vs. < 308), a larger body size (total length 265 mm vs. < 135 mm), a more gracile body (TL/BD = 76 vs. < 32), its tail proportion (TaiL/TD ratio 2.2 vs. < 1.2), its superior nasal suture (invisible dorsally vs. visible dorsally), more postoculars (2 vs. 1), and in its light-colored head cap (present vs. absent).

13) It differs from *I. meszoelyi* in its SIP (T-V vs. T-III), more postoculars (2 vs. 1), and its light-colored head cap (present vs. absent).

14) It differs from *I. pammeces* in its SIP (T-V vs. T-III), fewer midbody scale rows (18 vs. 20), its superior nasal suture (invisible dorsally vs. visible dorsally), and its light-colored head cap (present vs. absent).

15) It differs from *I. porrectus* in its anterior scale rows (18 vs. 20), more postoculars (2 vs. 1), and a light-colored head cap (present vs. absent), stout body (TL/BD = 78 vs. 86), more subcaudals (17 vs. 7-12).

16) It differs from *I. schmutzi* in having a larger body size (TL 265 mm vs. < 140 mm), more postoculars (2 vs. 1), and a light-colored head cap (present vs. absent).

17) It differs from *I. tenebrarum* in its SIP (T-V vs. T-III), fewer midbody scale rows (18 vs. 20), a larger body size (TL 265 mm vs. < 144 mm), its superior nasal suture (invisible dorsally vs. visible dorsally), more postoculars (2 vs. 1), and a light-colored head cap (present vs. absent).

18) It differs from *I. tenuicollis* in its SIP (T-V vs. T-III), fewer midbody scale rows (18 vs. 22), fewer total middorsals (432 vs. 480-523), rostral width (RW/HW = 0.35 vs. > 0.45), and its light-colored head cap (present vs. absent).

19) It differs from *I. veddae* in its SIP (T-V vs. T-III), fewer midbody scale rows (18 vs. 20), more total middorsals (432 vs. < 309), a larger body size (TL 265 mm vs. 93 mm), its tail proportion (TaiL/TD = 2.2 vs. 1.4), its superior nasal suture (invisible dorsally vs. visible dorsally), more postoculars (2 vs. 1), and a light-colored head cap (present vs. absent).

20) It differs from *I. violaceus* in having fewer midbody scale rows (18 vs. 20), more total middorsals (432 vs. < 308), a larger body size (TL 265 mm vs. < 135 mm), a thinner body width (TL/BD = 76 vs. < 43), its lateral snout profile (rounded vs. obtuse), its superior nasal suture (invisible dorsally vs. visible dorsally), its inferior nasal suture contact (preocular vs. 2^nd^ supralabial), more postoculars (2 vs. 1), and its light-colored head cap (present vs. absent).

##### Habitat and distribution.

The specimen was collected under a stone at the edge of a roadside forest near the China-Nepal border, which strongly suggests that this new species also occurs in Nepal. The sampling site is located within an evergreen broad-leaved forest, characterized by deep humus soil and a thick layer of leaf litter (Fig. 5). The sympatric herpetofauna at this locality includes *Protobothrops himalayanus*, *Orthriophis hodgsoni*, *Ablepharus mahabharatus*, and *Bufo himalayanus*.

##### Etymology.

The specific epithet *Gyirong* is a noun in apposition, referring to the type locality, Gyirong Town, Gyirong County, Xizang, China. We suggest the English common name “Gyirong blind snake” and the Chinese common name “吉隆盲蛇” (Jílóng Mángshé) for this new species.

## Discussion

Zhao (2006) and Wang et al. (2020) recognized five typhlopid blind snake species (from two genera) in China, with *Argyrophis diardii* and *Indotyphlops braminus* being the two species previously recorded from the QXP region (Yunnan Province, China) (Guo and Che 2024). The Gyirong blind snake is distinguished from *A. diardii* and its congener *I. braminus* by a combination of distinct external morphology (detailed above) and substantial genetic divergence (Fig. 2; Suppl. material 2). Additionally, while several *Indotyphlops* species, including *I. porrectus*, *I. braminus*, *I. jerdoni*, occur on the southern slopes of the QXP (India, Nepal, and Bhutan) (Schleich and Kastle 2002; Uetz et al. 2026), the new species is clearly different from these by both considerable genetic distance (Fig. 2) and a suite of unique morphological characters (the preceding comparisons). The description of this new species not only adds a new member to the global blind snake group, Typhlopidae, but also represents a new species to the Chinese herpetofauna. Importantly, it is the third blindsnake species reported from QXP and the first record of blind snakes in the Xizang Autonomous Region, China. This species was discovered near the border of China and Nepal, and it is highly likely to occur in Nepal, pending further field investigations.

SIP is widely recognized as a key diagnostic character for species identification within typhlopoid snakes (Pyron and Wallach 2014). Pyron and Wallach (2014) divided SIP into five states: T-I (first supralabial overlapping preocular), T-II (second supralabial overlapping preocular or presubocular), T-III (third supralabial overlapping ocular or subocular), T-V (both second and third supralabials overlapping shields above them), and T-0 (no overlapping supralabials). Within the superfamily Typhlopoidea, the T-III state is the dominant and most frequently observed SIP. The Gyirong blind snake is one of the exceptionally few documented *Indotyphlops* taxa that possess SIP T-V, a unique morphological feature that provides unambiguous diagnostic separation from the vast majority of its congeners, which exhibit SIP T-III (Pyron and Wallach 2014).

Previous studies on blind snakes are geographically fragmented and scattered, mainly due to their fossorial habits—which greatly increase the difficulty of field collection—and relatively conserved morphology, which hinders accurate morphological identification and species delimitation. Consequently, comprehensive systematic investigations are scarce, with most studies confined to specific geographic regions and relying on a limited set of specimens and characters (McDowell 1974; Roux-Estève 1974; Rabosky et al. 2004; Broadley and Wallach 2009). Additionally, most blind snake species lack sufficient genetic data. This leaves many key phylogenetic relationships within the group unresolved. Resolving these issues will depend on extensive international collaboration and the large-scale use of genomic data, both of which are essential for establishing a more complete and robust evolutionary framework.

In recent years, with the extensive field exploration being conducted in Xizang, China, many new taxa or new national records have been recorded, particularly on the borders between China and its neighbors such as India, Nepal and Bhutan (Che et al. 2020; Guo et al. 2024, 2025; Shu et al. 2024; Wang et al. 2025; Tan et al. 2026), indicating these regions need further investigation.

## Supplementary Material

XML Treatment for Indotyphlops gyirongensis

## Additional information

### Artificial Intelligence (AI) use

The authors accept full responsibility for the content of the manuscript, including the disclosure of any use of AI.

No AI tools were used in the preparation of this manuscript.

### Author ORCIDs

B. Lyu https://orcid.org/0000-0001-5594-1543

S. Tan https://orcid.org/0009-0009-1461-6328

Z. Zhao https://orcid.org/0009-0002-7716-6255

V. Wallach https://orcid.org/0000-0003-0844-045X

Q. Liu https://orcid.org/0000-0001-8402-6491

P. Guo https://orcid.org/0000-0001-5585-292X

### Data availability

All of the data that support the findings of this study are available in the main text or Supplementary Information.

## Supplementary materials

Supplementary material 1 Information for the samples Data type xlsx Explanation note Detailed information for the samples used in this study. https://binary.pensoft.net/file/1776843This dataset is made available under the Open Database License (http://opendatacommons.org/licenses/odbl/1.0/). The Open Database License (ODbL) is a license agreement intended to allow users to freely share, modify, and use this Dataset while maintaining this same freedom for others, provided that the original source and author(s) are credited. Bing Lyu, Songwen Tan, Zhichao Zhao, Van Wallach, Qin Liu, Peng Guo

Supplementary material 2 Uncorrected p-distance based on Cytb sequences Data type xlsx Explanation note Uncorrected p-distance between species based on Cytb sequences (standard error estimates are shown above the diagonal). https://binary.pensoft.net/file/1776844This dataset is made available under the Open Database License (http://opendatacommons.org/licenses/odbl/1.0/). The Open Database License (ODbL) is a license agreement intended to allow users to freely share, modify, and use this Dataset while maintaining this same freedom for others, provided that the original source and author(s) are credited. Bing Lyu, Songwen Tan, Zhichao Zhao, Van Wallach, Qin Liu, Peng Guo

Supplementary material 3 Uncorrected p-distance based on CO1 sequences Data type xlsx Explanation note Uncorrected p-distance between species based on CO1 sequences (standard error estimates are shown above the diagonal). https://binary.pensoft.net/file/1776845This dataset is made available under the Open Database License (http://opendatacommons.org/licenses/odbl/1.0/). The Open Database License (ODbL) is a license agreement intended to allow users to freely share, modify, and use this Dataset while maintaining this same freedom for others, provided that the original source and author(s) are credited. Bing Lyu, Songwen Tan, Zhichao Zhao, Van Wallach, Qin Liu, Peng Guo
